# Polarized Secretion of APRIL by the Tonsil Epithelium Upon Toll-Like Receptor Stimulation

**DOI:** 10.3389/fimmu.2021.715724

**Published:** 2021-08-18

**Authors:** Nathalie Sturm, Melanie Quinterot, Jean-Philippe Guyot, Christian Righini, Willeke F. Daamen, Toin H. van Kuppevelt, Bertrand Huard

**Affiliations:** ^1^Department of Pathology, University Hospital, Grenoble, France; ^2^Translational Innovation in Medicine and Complexity, Centre National de la Recherche Scientifique UMR5525, La Tronche, France; ^3^Oto-Rhino-Laryngology Unit, University Hospital, Geneva, Switzerland; ^4^Head and Neck Department, University Hospital, Grenoble, France; ^5^Department of Biochemistry, Radboud Institute for Molecular Life Sciences, University Medical Center, Nijmegen, Netherlands

**Keywords:** mucosa, plasmocytes, survival, epithelium, APRIL

## Abstract

In mucosa such as tonsil, antibody-producing plasmocytes (PCs) lie in sub-epithelium space, which is thought to provide a suitable environment for their survival. A proliferation inducing ligand (APRIL) is one key survival factor for PCs present in this area. According to *in situ* staining, apical epithelial cells produced APRIL, and the secreted product had to migrate all through the stratified surface epithelium to reach basal cells. A similar process also occurred in the less-organized crypt epithelium. Tonsil epithelial cells captured secreted APRIL, thanks to their surface expression of the APRIL coreceptor, either syndecan-1 or -4 depending on their differentiation stage. In the most basal epithelial cells, secreted APRIL accumulated inside secretory lamp-1^+^ vesicles in a polarized manner, facing the sub-epithelium. The tonsil epithelium upregulated APRIL production by apical cells and secretion by basal cells upon Toll-like receptor stimulation. Furthermore, LPS-stimulated epithelial cells sustained *in vitro* PC survival in a secreted APRIL-dependent manner. Taken together, our study shows that the tonsil epithelium responds to pathogen sensing by a polarized secretion of APRIL in the sub-epithelial space, wherein PCs reside.

## Introduction

A proliferation inducing ligand (APRIL) is one of the last cloned members of the tumor necrosis factor superfamily (TNFSF13) (1). APRIL has two canonical signaling receptors from the TNF receptor superfamily, the B-cell maturation antigen (BCMA, TNFRSF18) and the transmembrane activator and CAML interactor (TACI, TNFRSF13B). These receptors are acquired at specific differentiation stages after B-cell encounter with their cognate antigens. The latter explains the late role of APRIL in humoral immunity, mostly in the generation and survival of antibody-producing plasmocytes (PCs) (2). APRIL is first produced as a transmembrane type II protein, and cleaved early in the exocytic pathway in the Golgi apparatus by furin proteases (3). As a consequence, full-length APRIL is not present at the surface of producing cells. Quite unique in the TNFSF, APRIL, once secreted as a trimer, requires the coreceptor activity of heparan sulfate proteoglycans (HSPG) such as syndecan molecules to efficiently signal into target cells (4, 5). HSPG are proteins containing an O-linked heparan chain constituted by repeats of the disaccharide unit N-acetyl glucosamine/glucuronic acid onto which sulfate residues are added. The ligand specificity of HSPG heavily relies on their sulfation pattern generated by the maturation action of different sulfotransferases (6). Specific positioning of negatively charged sulfate residues on the heparan chain determines the binding ability to proteins. These proteins must contain a stretch of positively charged basic residues, such as the one accessible in the N-terminus of APRIL after furin processing.

Myeloid cells including macrophages, dendritic cells, eosinophils, and neutrophils are the main cellular source of APRIL (7). In some cells, i.e., neutrophils and their bone marrow precursors, APRIL production is constitutive, at least in part (8, 9). This implies a constant and high steady state level of APRIL in the bone marrow, an organ known to host long-lived PCs. This also implies that any neutrophil extravasation will provide APRIL in an inflamed tissue. Epithelial cells also produce APRIL (10, 11). Here, we are showing in tonsils that a polarized secretion of APRIL by apical epithelial cells upon TLR stimulation occurs across this stratified epithelium to deliver APRIL to sub-epithelial PCs.

## Material and Methods

### Human Samples

Samples were obtained at the ORL department of the Grenoble-Alpes and Geneva University Hospitals from patients with acute tonsillitis and recurrent tonsillitis associated to infections and from patients with snoring problems from 2008 to 2015. For the latter, the patients had not experienced any tonsil infections for the previous 2 years and had not been treated with antibiotics during the last 3 months preceding surgery. Patients gave their informed consent.

### Immunohistochemistry

Stalk-1 (rabbit polyclonal) detecting APRIL-producing cells, Aprily-2 (mouse IgG1) detecting secreted APRI, 10e4 (mouse IgM, AMS-Bio) detecting native heparan sulfate (HS) chains, 3g10 (mouse IgG2b, AMS-Bio) detecting the HS stub after heparitinase treatment, and the VSV G tagged ScFv LKiV69 and HS4E4 recognizing N-/2-O sulfates and N- sulfates, respectively, have been previously described (12–15). Formalin-fixed paraffin-embedded tonsil sections of 3 µm were stained with these antibodies. Detection was based on a horseradish peroxidase–labeled polymer conjugated with secondary antibodies (EnVision^®^+, Agilent). Development was done using 3amino-9-ethyl-carbazol (Sigma-Aldrich), and sections were counterstained with Mayer’s hematoxylin (Agilent). Antibodies against syndecan-4 (citrate-based antigen retrieval, rabbit polyclonal, SC15350, Santa Cruz Biotechnology) and syndecan-1 (citrate-based antigen retrieval, rabbit polyclonal, 362900, Life Technologies) were used at 10 µg/ml after a heat-induced epitope retrieval treatment in citrate buffer pH 6. For 58K (clone 58K9, mouse IgG1 Abcam) and lamp-1 (N19, goat IgG, Santa Cruz Biotechnology), staining was performed on frozen sections of tonsils. To circumvent the problem of identical isotype between 58k9 and Aprily-2, the 58K9 staining was first revealed with a fluorochrome-conjugated anti-mouse IgG1 followed by the staining of biotinylated Aprily-2 in the presence of excess of irrelevant mouse IgG1. Alexa-488 and phycoerythrin-conjugated species/isotype specific secondary antibodies and streptavidin were all from Life technologies. DAPI (Agilent) was used for nucleus staining. Peroxidase-based stainings were visualized with an Axiophot 1, captured with an Axiocam color CDD camera, and treated on a Pentium III computer with the AxioVision software, all from Carl Zeiss AG. For PC numeration, image acquisition was done at the ×40 objective, and syndecan-1-stained cells were counted in the entire field corresponding to a surface of 30 mm^2^. For secreted APRIL signal quantification, images acquired with the 40× objective were processed with the metamorph image analysis software (Molecular devices). Color threshold was selected in the hue saturation intensity space. Saturation values obtained in the threshold field were added, logged to a spreadsheet, and expressed in arbitrary unit. Fluorescent costainings were visualized by confocal microscopy with a LSM510 (Carl Zeiss). Secreted APRIL/syndecan-4 colocalization was analyzed by using “RG2B colocalization” plugin for ImageJ. Auto thresholding was used and pixel intensity acquired with measure tool for processed and original images. Ratio between the two measures determined colocalization percentage. Quantification was performed on 90 cells from three independent tonsils.

### Flow Cytometry

The 293-T cells were transiently transfected by calcium phosphate DNA precipitation as previously described (12). Plasmids encoding for syndecan-1, -2, and -4 were kindly provided by Dr. Pascal Schneider (University of Lausanne, Switzerland) (4). Three days after transfection, adherent cells were harvested with PBS containing 5 mM EDTA and stained with 10 µg/ml of rabbit polyclonal anti-syndecan-4 and syndecan-1 antibodies. Detection was performed with phycoerythrin-conjugated goat anti-rabbit antibodies. Fluorescence was analyzed on a FACSCalibur (BD Biosciences). FACS-sorting of CD19^+^ CD38^high^ tonsil plasmocytes was performed as previously described (10).

### Quantitative RT-PCR

Total RNAs were prepared from tonsil samples using Trizol (Thermo Fisher Scientific), treated with RQ1 DNAse (Promega), and reverse transcribed with Superscript II reverse transcriptase and oligo-dT_12-18_ (Thermo Fisher). Quantitative RT-PCR was done with a light cycler System (Roche Diagnostics) and the Quantitect SYBR Green PCR kit solution (Qiagen). For hAPRIL, 5ʹ-ATGGGTCAGGTGGTGTCTCG-3ʹ and 5ʹ-ATGGGTCAGGTGGTGTCTCG-3ʹ were used as forward and reverse primers, respectively. The control GAPDH primers were 5ʹ-TCATGGGTGTGAACCATGAGA-3ʹ (forward) and 5ʹ-GCTAAGCAGTTGGTGGTGCA-3ʹ (reverse). APRIL cDNA was used to establish the APRIL standard curve. Total reverse transcribed RNA from Hela cells was used for the GAPDH standard curve. Results were normalized to GAPDH and expressed as arbitrary units. Amplification was done in duplicates, and experiments were performed twice.

### ELISA

Each tonsil was cut in four slices to perform simulations in medium alone, 100 ng/ml of LPS (Sigma), 100 ng/ml of LPS with 5 µM actinomycin, and 100 ng/ml of LPS +100 nM tetanus toxin for 10 h. Tonsils were also stimulated the same way with 10 µg/ml of peptidoglycan (Sigma). Medium was RPMI-1640 medium (Gibco) containing 10% fetal calf serum (Eurobio) and penicillin/streptomycin (Gibco). After stimulation, specific parts of tonsils separated under a binocular loop were pooled and lyzed in Na phosphate 0.01 M, EDTA 1 mM, EGTA 1 mM, NaF 1 mM, NaCl 0.15 M pH=7.2, containing 1% Triton X-100, 0.1% NaDOC, 1 mM PMSF, and complete mini cocktail protease inhibitors from Roche. Protein quantification in lysates was performed with a BCA protein assay kit (Pierce). To obtain conditioned supernatants from HaCat cells, seeding was performed at 0,1 10e^6^ cells/ml, and supernatants were harvested 2 days after stimulation. APRIL protein concentration was measured with the APRIL ELISA kit from Bender Medsystems.

### *In Vitro* PC Survival

HaCat cells obtained at the American tissue culture collection were seeded at a concentration of 0,1 10e^6^ cells per ml in the upper compartment of 0,4 µm Transwell plates (Corning), and stimulated for 2 days with 100 ng/ml of LPS. Then 0,1 10e^6^ purified tonsil plasmocytes cells were seeded in the bottom compartment. The blocking antibody to human APRIL (Mahya-1, mouse IgG1, Adipogen) was used at 10 µg/ml. Live cells were numerated by trypan blue exclusion.

### Statistics

Statistical analysis was performed using GraphPad Prism software. Normality of data set distribution was tested with the D’Agostino and Pearson test. Parametric and non-parametric t-tests were used to compare two data sets with and without, respectively, normal distributions. For multiple group analysis, parametric and non-parametric ANOVA tests were run. Correlation between two data sets were analyzed with the Pearson’s *r* coefficient for normally distributed data sets. Significant differences were defined as p < 0.05. *p < 0.05, **p < 0.01, ***p < 0.001, ****p < 0.0001.

## Results

### APRIL Produced by Apical Epithelial Cells Is Translocated at the Basal Face

**Figure 1:**
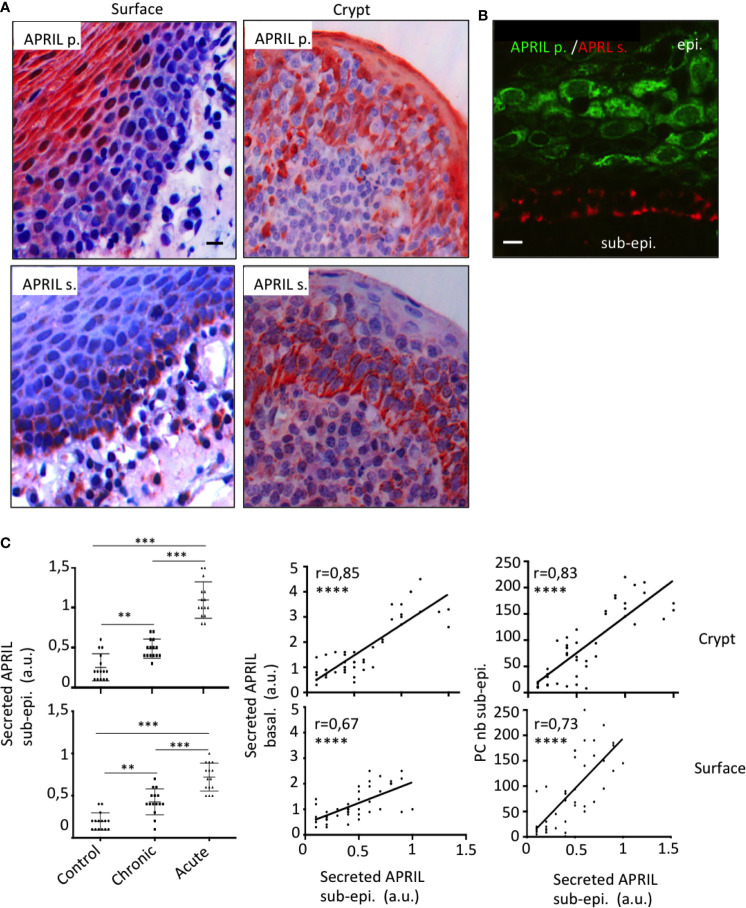
Production and storage sites for APRIL are distant in the tonsil epithelium. **(A)** Serial sections of tonsils were stained for APRIL production (APRIL p.) and secreted APRIL (APRIL s.). Scale bar = 10 µm. **(B)** Costaining for APRIL p. (green) and APRIL s. (red) on the same section is shown. Scale bar = 5 µm. The epithelium (epi.) and sub-epithelium (sub-epi.) are marked. Results are representative of more than 15 tonsillectomies for patients with chronic infections. **(C)** Staining quantification for 15 control, 15 chronically infected, and 15 acutely infected tonsils is shown for secreted APRIL in the sub-epithelial area (left panel). A one-way unpaired parametric ANOVA test was performed. Correlations with the staining intensity obtained in the basal layer of the epithelium (middle panel) and density of PCs in the area (right panel) are also shown. A Pearson’s correlation *t* test was performed. **p < 0.01, ***p < 0.001, ****p < 0.0001.

In the stratified epithelium from tonsil surface, several layers of the apical face were reactive with the antibody detecting APRIL-producing cells (**Figure 1A**). Notably, processed APRIL accumulated at distance of its production site in the most basal layer of this surface epithelium. The crypt epithelium from tonsil showed a similar expression pattern although less pronounced between the apical and basal surfaces. Some secreted APRIL was also detected in the sub-epithelial zone from both areas. Costaining confirmed the distance between the production and storage sites for APRIL in the tonsil epithelium (**Figure 1B**). We observed a similar expression pattern for APRIL production and secreted APRIL with other stratified epithelia from mouth skin (**Figure Sup1**). In the latter, inflammatory conditions such as subcutaneous melanoma development were associated to APRIL production in apical layers and retention of the secreted product in the basal layer. By contrast, non-inflammatory normal skin revealed a complete absence of APRIL, indicating an inflammatory component regulating its production. We previously observed that the intensity of Stalk-1 staining did not vary in apical epithelial cells from control and chronically and acutely infected tonsils (10). Similarly to what we showed in this latter study for the whole intensity of secreted APRIL, the intensity for secreted APRIL measured in the sub-epithelial area from crypts significantly increased from control to chronic and from chronic to acute tonsils (**Figure 1C**, upper panels). Notably, the intensity of secreted APRIL in this sub-epithelial area correlated with the intensity recorded in the basal face of this epithelium. In details, the intensity in the basal layer was significantly higher than in the sub-epithelial area (mean +/− SD: 1.7 +/− 1,1 *vs* 0.6 +/− 0,3, p<0,0001, paired parametric *t* test). The latter may indicate an accumulation step in basal cells. Finally, the intensity of secreted APRIL in the sub-epithelial area correlated with the number of crypt PCs also numerated in this area. Similar results were obtained when we analyzed all these parameters for the surface epithelium (**Figure 1C**, bottom panels). When the surface and crypt epithelia were compared, values for secreted APRIL were significantly higher in the crypt, while PC number was not different (**Figure Sup2**). Taken together, this indicates that APRIL, once produced and processed, migrates from the apical layers to the most basal layer in the stratified epithelia. Our data also indicate that some secreted APRIL are delivered to the sub-epithelial area. The positive correlation found between the density of secreted APRIL and PC strongly suggests that APRIL plays a survival role for PCs at that place.

### Expression of Heparan Sulfate Proteoglycan in the Tonsil Epithelium

**Figure 2:**
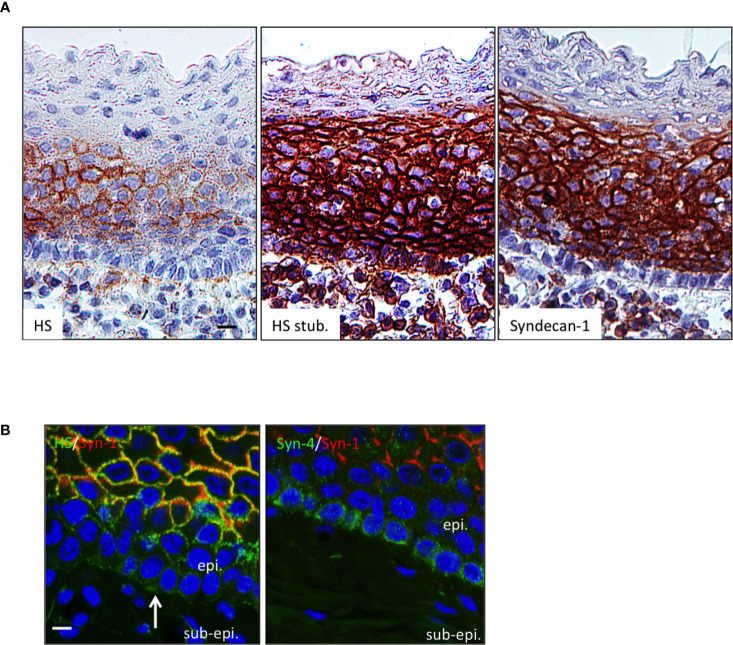
The HSPG syndecan-1 and -4 are expressed by tonsil epithelial cells. **(A)** Serial sections of tonsils were stained for native HS chain (10e4 reactivity), the HS stub (3g10 reactivity), and syndecan-1. Scale bar = 10 µm. **(B)** Tonsil sections were costained for 10e4 (green)/syndecan-1 (red) and syndecan-4 (green)/syndecan-1 (red). Scale bar = 5 µm. The surface epithelium is shown. In some figures, the epithelium (epi.) and sub-epithelium (sub-epi.) are marked. The arrow and box show the basement membrane and a plasmocyte, respectively. Results are representative of more than 15 tonsillectomies for patients with recurrent infections.

APRIL is commonly scavenged at the surface of target cells by HSPG. The surface tonsil epithelium harbored surface HS chain as revealed in native conditions with the 10e4 antibody. The 10e4 reacted with middle layers of the epithelium but not with the most basal one (**Figure 2A**). The 10e4 also stained the basement membrane between the basal layer and sub-epithelium. The most basal layer harbored HS of different nature only stained with the stub-reactive 3g10 antibody. This basal layer expressed a maturated HS chain reactive with the other anti-native HS LKiV69 and HS4E4 antibodies (**Figure Sup3**). To determine the proteic core of epithelial HSPG, we used polyclonal antibodies against syndecans. Flow cytometry on transfected cells demonstrated that these polyclonal antibodies were member-specific in the syndecan family (**Figure Sup4**). The anti-syndecan-1 stained the surface of epithelial cells from middle layers but not the extreme basal layer constituted by less differentiated cells. Costaining showed that syndecan-1 was 10e4 reactive on epithelial cells from middle layers. We further identified syndecan-4 on the surface of basal cells. The anti-syndecan-1 also detected sub-epithelial PCs harboring a classical eccentric nucleus. Syndecan-1 on plasmocytes also harbored HS chains as revealed by their reactivity with 3g10 and to a lesser extent 10e4 (see inserts in **Figure 2**). Taken together, this shows that the APRIL-coreceptor HSPGs are widely expressed by tonsil epithelial cells with different proteic cores and mature HS chains.

### Basal Epithelial Cells Store APRIL in a Lamp-1^+^ Compartment

**Figure 3:**
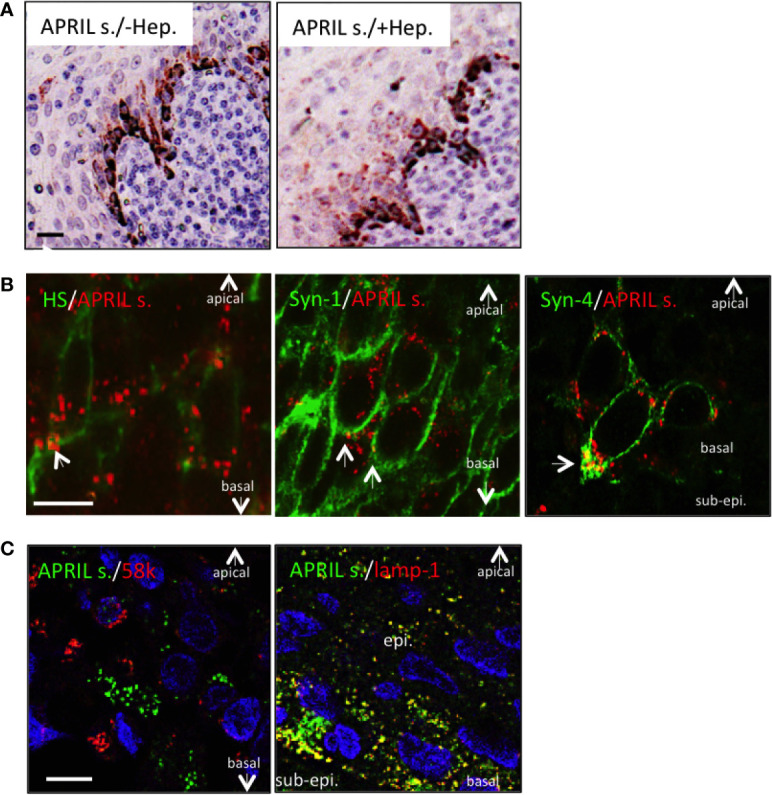
Processed APRIL accumulates in LAMP-1+ vesicles in the basal layer. **(A)** Serial sections of tonsils were stained for secreted APRIL in the absence or presence of heparitinase treatment. Scale bar = 20 µm. Results are representative of three tonsillectomies. **(B)** Secreted APRIL (red) was costained with syndecan-1/-4 and native HS (all green). Arrows show colocalization of secreted APRIL with HS/syndecan-1/syndecan-4. Scale bar = 5 µm. Results are representative of 10 tonsillectomies. **(C)** Secreted APRIL (green) was costained with Golgi (58k, red) and exosome (Lamp-1, green) markers. Scale bar = 10 µm. Surface epithelia are shown with the apical, basal, and sub-epithelium (sub-epi.) localizations marked. In some experiments, nuclear staining (DAPI) is shown. Patients with recurrent infections were used. Results are representative of 10 tonsillectomies.

We next wanted to determine the fine localization of secreted APRIL in the tonsil epithelium. We first noticed that heparitinase treatment did not significantly diminish secreted APRIL reactivity in epithelial cells (**Figure 3A**). This indicated that secreted APRIL is predominantly not bound to HSPG. Costaining experiments confirmed that only a minority of secreted APRIL detected at the surface of epithelial cells colocalized with native HS and syndecan-1 (**Figure 3B**, arrows). Most of the secreted APRIL resided intracellularly in a HS, syndecan-1, and syndecan-4 negative compartment both in middle layers and the most basal layer. Notably, in the syndecan-4 most basal layer, compartments rich in secreted APRIL polarized to the basal face. There, we measured a colocalization of 23,4% +/− 5 between secreted APRIL and syndecan-4. Finally, use of organelle-specific markers showed that most (70% +/− 15) secreted APRIL resided in lamp1^+^ positive cells (**Figure 3C**). No secreted APRIL resided in 58K-reactive organelles from the Golgi apparatus. Accumulation in lamp-1^+^ vesicles was particularly evident in basal cells. Taken together, this indicates that epithelial cells capture APRIL by their surface syndecan, and APRIL migrates across the different layers of epithelial cells in intracellular lamp-1^+^ vesicles. In the basal layer, vesicles rich in secreted APRIL show a polarized localization towards the sub-epithelial space.

### Toll-Like Receptor Stimulation Upregulates APRIL Delivery to the Sub-epithelial Zone to Improve Plasmocyte Survival

**Figure 4:**
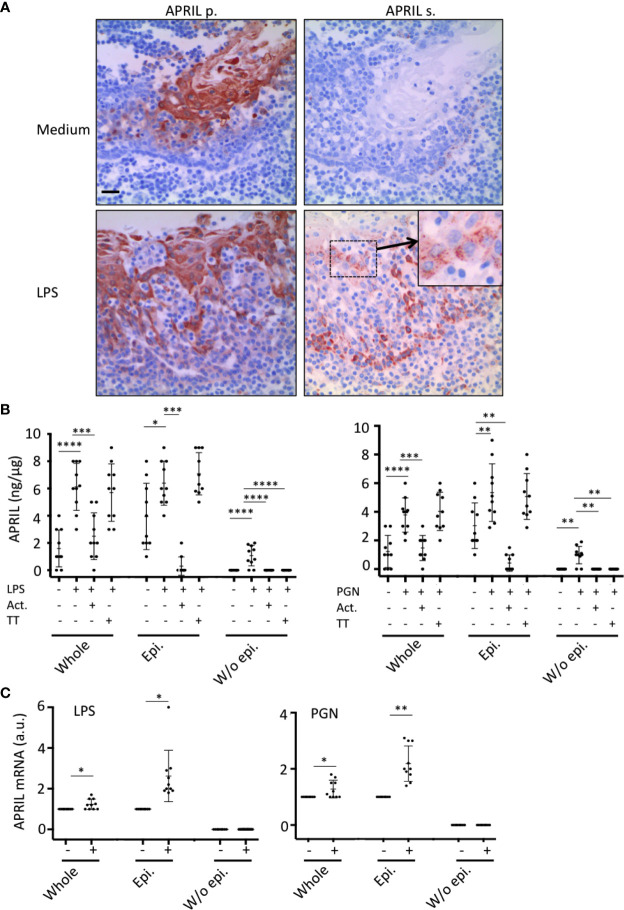
Tonsil epithelium secretes APRIL in the parenchyma upon TLR stimulation. **(A)** Serial sections of medium- and LPS-stimulated tonsils were stained for APRIL production and secreted APRIL. Insert shows a high magnification of a cluster of apical epithelial cells positive for secreted APRIL. A crypt epithelium is shown. Scale bar = 20 µm. **(B)** APRIL was quantified by ELISA from the indicated parts of unstimulated, LPS-, and PGN-stimulated tonsils. Actinomycin D (Act.) and Tetanus toxin (TT) were used to block transcription and exocytosis, respectively. A paired parametric ANOVA test was performed. **(C)** APRIL mRNA was quantified by qRT-PCR in the indicated part of unstimulated and LPS/PGN-stimulated tonsils. Results are representative of 10 tonsillectomies. Non-inflamed tonsils removed for snoring problems were used. Means +/− SD are shown. A paired non-parametric *t* test was performed. *p < 0.05, **p < 0.01, ***p < 0.001, ****p < 0.0001.

**Figure 5:**
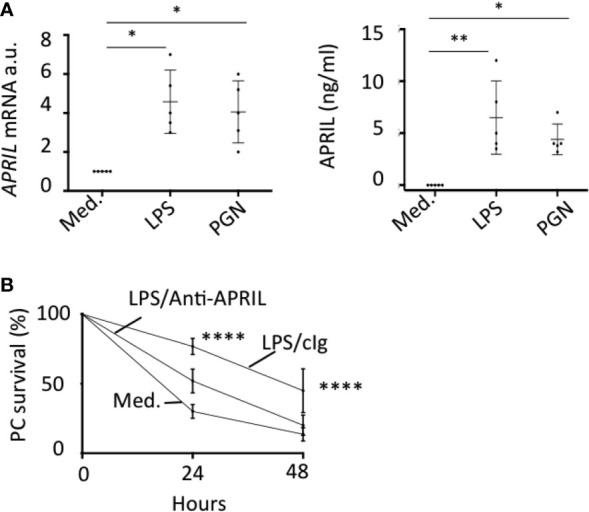
TLR-stimulated APRIL production by epithelial cells sustains plasmocyte survival. **(A)** HaCat cells were stimulated with LPS and PGN. APRIL mRNA (left panel) and protein in the cell supernatant (right panel) were quantified by qRT-PCR and ELISA, respectively, after 48 h. Five independent experiments were performed. One-way non-parametric (mRNA) and parametric (protein) ANOVA tests were performed. **(B)** HaCat cells were seeded in the upper compartment of a transwell and stimulated with 100 ng/ml of LPS for 48 h. FACS-sorted tonsil PCs were added in the bottom compartment. The % of PC survival at the indicated time is shown with 100% defined by the initial PC input. Means +/− SD are shown. An unpaired parametric two-way ANOVA test was performed. *p < 0.05, **p < 0.01, ****p < 0.0001.

*In situ* staining right after tonsillectomy never allowed us to detect full-length APRIL defined by a colocalization in apical epithelial cells between the reactivity of the antibodies detecting APRIL-producing cells and secreted APRIL. This indicated a rapid processing by furin proteases and transport of the secreted product in adjacent epithelial cells. A short *in vitro* stimulation of whole tonsils by LPS allowed us to observe secreted APRIL reactivity in apical layers in addition to the accumulation at the basal layer (**Figure 4A**). In this setting, unstimulated tonsils kept the APRIL production staining in apical cells most likely due to the stability of the processed fragment in producing cells, and showed much less secreted APRIL accumulation in basal layers. This *in vitro* short-term tonsil culture allowed the detection of the APRIL protein by ELISA with lysed samples of whole tonsils (**Figure 4B**). We next performed macroscopic dissection of tonsils to separate the epithelium from the sub-epithelial zone. Without stimulation, we only detected weak quantities of APRIL in fractions containing epithelial cells. LPS stimulation largely increased APRIL detection in whole and epithelial lysates. In this condition, the sub-epithelial fraction was also positive. Notably, use of Actinomycin D to block new transcription significantly downregulated APRIL concentration in all samples. Use of tetanus toxin (TT) is known to block exocytosis (16). TT only diminished APRIL concentration in sub-epithelial lysates. Similar results were reproduced when tonsils were stimulated with PGN. Analysis at the mRNA level by qRT-PCR showed that production was only detected in samples containing epithelial cells and that this transcription was upregulated by LPS and PGN stimulation (**Figure 4C**). We next used the HaCat keratinocyte cell line as an *in vitro* model of epithelial cells. LPS and PGN stimulation upregulated APRIL transcription and secretion in these cells (**Figure 5A**). Notably, APRIL secreted by HaCat cells significantly sustained the *in vitro* survival of purified tonsil PC (**Figure 5B**). Taken together, these showed that APRIL production in epithelial cells is not constitutive but dependent of TLR stimulation, at least in part. Notably, pathogen sensing by TLR from tonsil epithelial cells upregulated secreted APRIL delivery to induce PC survival.

## Discussion

An epithelium formed with tight junctions serves in immunity as a physical barrier (17). In addition, epithelial cells are active immune players by the secretion of molecules such as antimicrobial peptides, chemokines, and cytokines. They also transport antibodies in a polarized transport-dependent manner (18). The best-known example of the latter is the basal to apical transport of IgA taken up from the lamina propria to the lumen of the intestine. This process is mediated by a specific receptor, the polymeric IgA receptor. The reversed polarized transport from apical to basal across an epithelium also occurs. This is the case for the delivery of maternal IgG to the fetus *via* the neonatal Fc receptor. Here, we are showing that an apical to basal polarized secretion pathway also exists for APRIL. This process is making a lot of sense, since only apical epithelial cells from a stratified epithelium with tight junctions may sense pathogens, and the APRIL target cell, the plasmocyte, is known to reside beneath the epithelium, in the sub-epithelial area.

Tonsil epithelial cells express at their surface the HSPG syndecan-1 and syndecan-4. Syndecan-1 is expressed in middle layers but not the most basal one, which expresses syndecan-4. This demonstrates proteic core variability among HSPG according to epithelial cell differentiation stage. Another variability exists at the level of the HS chain maturation. The 3g10 reactivity considered as being close to a pan HSPG was widely present in middle and basal layers. The 10e4 antibody recognizes HS chains containing N-unsubstituted glucosamine (19). The 10e4 reacts only with middle layers but not basal ones. The latter do possess a mature HS chain stained by the LKIV69 and HS4E4 antibodies, recognizing at least N-/2-O sulfates and N- sulfates, respectively. These two antibodies also stained epithelial cells above the most basal layer. Taken together, this shows the high variable HSPG content at the surface of epithelial cells, at least in the tonsil epithelium. We did not find evidences for syndecan/HS surface expression by most apical epithelial cells very certainly to avoid the recapture of the APRIL they secrete. Notably, syndecans have already been implicated in the apical to basal transport in the case of human immunodeficient virus through the genital epithelium (20). In the tonsil epithelium, secreted APRIL was mainly detected intracellularly in lamp-1^+^ vesicles devoid of syndecan expression. In fact, APRIL transport across the tonsil epithelium engages an intracellular route with lamp-1^+^ vesicles. Notably, lamp-1 vesicles have already been implicated in an apical to basal transport in endothelial cells (21). Hence, it is likely that the polarized secretion of APRIL uses such a pathway to cross the tonsil epithelium. The predominant intracellular localization of secreted APRIL observed here in all the tonsils analyzed does not mean that APRIL transport is independent of HS and syndecans. Indeed, detection of secreted APRIL at the surface of epithelial cells bound to HS/syndecan was possible even though to a much lower extent than its intracellular localization. Hence, epithelial cells may capture APRIL on to their surface syndecan before translocating it *via* intracellular lamp-1^+^ vesicles. Our study has some limitations, since the secretion pathway has been studied in fixed materials in the absence of any kinetics experiments. We also did not use for *in vitro* experiments a stratified epithelium to deeply assess the cellular pathways implicated in these processes.

Tonsil epithelial cells with the use of cell lines have been previously shown to respond to TLR ligands (22). Here, we are showing that production of APRIL by apical epithelial cells from tonsils is largely upregulated by peptidoglycan, a TLR-2 ligand, and LPS, a TLR-4 ligand. In addition, TLR stimulation also increases APRIL secretion in the sub-epithelial zone. We observed a similar pattern of APRIL expression in other stratified epithelium such as the mouth and the skin, indicating that APRIL polarized secretion may occur in different stratified epithelium, keratinizing or not. Our present study highlights a translocation process of APRIL produced by apical cells across both tonsil epithelium, surface and crypt, with a delivery to the sub-epithelial area. Further investigations are required to decipher the cellular pathways mediating such polarized secretion. Owing to the well-characterized prosurvival role of APRIL for PC, it is very much likely that this process serves to maintain PCs produced in this secondary lymphoid organ. Notably, the entire process, from APRIL production by apical cells to the delivery by basal cells to the sub-epithelium, is upregulated in the case of an infection. Hence, PC survival in the tonsil should be largely dependent on the inflammation status of the organ, a concept that has been already postulated for PC survival outside the bone marrow (23).

## Data Availability Statement

The original contributions presented in the study are included in the article/**Supplementary Material**. Further inquiries can be directed to the corresponding author.

## Ethics Statement

The studies involving human participants were reviewed and approved by Geneva ethics committee for human studies/Grenoble ethics committee for human studies. The ethics committee waived the requirement of written informed consent for participation.

## Author Contributions

J-PG and CR provided tonsillectomies. DW and TK analyzed the anti-HS reactivities. NS and MQ performed and analyzed immunohistochemistry. BH designed the study, performed experiments, analyzed data, and wrote the article. All authors contributed to the article and approved the submitted version.

## Funding

This work was supported by a grant from the FINOVI foundation to BH and the AFEF to NS.

## Conflict of Interest

The authors declare that the research was conducted in the absence of any commercial or financial relationships that could be construed as a potential conflict of interest.

## Publisher’s Note

All claims expressed in this article are solely those of the authors and do not necessarily represent those of their affiliated organizations, or those of the publisher, the editors and the reviewers. Any product that may be evaluated in this article, or claim that may be made by its manufacturer, is not guaranteed or endorsed by the publisher.
