# Supplementary material for: Polarized Secretion of APRIL by the Tonsil Epithelium Upon Toll-Like Receptor Stimulation
Source: Front Immunol. 2021 Aug 18;12:715724. doi: 10.3389/fimmu.2021.715724 (PMC8416415; doi:10.3389/fimmu.2021.715724)
Supplement: Supplementary file 1 [file DataSheet_1.docx]

Supplementary Material

## Supplementary Figures

**
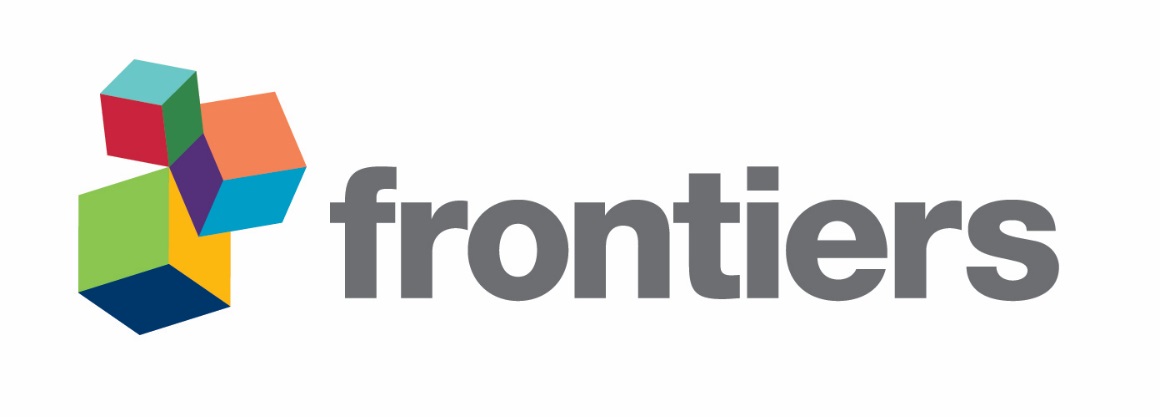
**

**Figure 1sup: epithelial production and storage sites for APRIL in different tissues**

Serial sections of the indicated epithelia were stained for APRIL production and secreted APRIL. Scale bar = 20 µm. Results are representative of at least five cases per epithelium. Brownish stain in the normal skin corresponds to melanin pigment.

**Figure 2sup: APRIL secretion and PC density for the surface and crypt epithelium**

Staining for secreted APRIL and PC number were quantified and compared between the crypt and surface epithelium. A paired parametric t test was performed. ***: p<0,001, ****:p<0,0001.

**Figure 3sup: reactivity of the basal epithelial cells with anti-HS antibodies.**

Serial sections of a tonsil epithelium were stained with the indicated anti-HS. Scale bar = 10 µm

Results are representative of ten tonsils.

**Figure 4sup: fine specifity of anti-syndecan antibodies**

293-T cells transiently transfected with plasmids encoding for syndecan-1, -2 and -4 were stained with the indicated antibodies and analyzed by flow cytometry. Results are representative of two independent experiments.
